# RT-qPCR reveals opsin gene upregulation associated with age and sex in guppies (*Poecilia reticulata*) - a species with color-based sexual selection and 11 visual-opsin genes

**DOI:** 10.1186/1471-2148-11-81

**Published:** 2011-03-29

**Authors:** Christopher RJ Laver, John S Taylor

**Affiliations:** 1 University of Victoria, Department of Biology, Victoria, British Columbia, Canada

## Abstract

**Background:**

PCR-based surveys have shown that guppies (*Poecilia reticulata*) have an unusually large visual-opsin gene repertoire. This has led to speculation that opsin duplication and divergence has enhanced the evolution of elaborate male coloration because it improves spectral sensitivity and/or discrimination in females. However, this conjecture on evolutionary connections between opsin repertoire, vision, mate choice, and male coloration was generated with little data on gene expression. Here, we used RT-qPCR to survey visual-opsin gene expression in the eyes of males, females, and juveniles in order to further understand color-based sexual selection from the perspective of the visual system.

**Results:**

Juvenile and adult (male and female) guppies express 10 visual opsins at varying levels in the eye. Two opsin genes in juveniles, *SWS2B *and *RH2-2*, accounted for >85% of all visual-opsin transcripts in the eye, excluding *RH1*. This relative abundance (RA) value dropped to about 65% in adults, as *LWS-A180 *expression increased from approximately 3% to 20% RA. The juvenile-to-female transition also showed *LWS-S180 *upregulation from about 1.5% to 7% RA. Finally, we found that expression in guppies' *SWS2-LWS *gene cluster is negatively correlated with distance from a candidate locus control region (LCR).

**Conclusions:**

Selective pressures influencing visual-opsin gene expression appear to differ among age and sex. *LWS *upregulation in females is implicated in augmenting spectral discrimination of male coloration and courtship displays. In males, enhanced discrimination of carotenoid-rich food and possibly rival males are strong candidate selective pressures driving *LWS *upregulation. These developmental changes in expression suggest that adults possess better wavelength discrimination than juveniles. Opsin expression within the *SWS2-LWS *gene cluster appears to be regulated, in part, by a common LCR. Finally, by comparing our RT-qPCR data to MSP data, we were able to propose the first opsin-to-λ_max _assignments for all photoreceptor types in the cone mosaic.

## Background

For the better part of a century, guppies (*Poecilia reticulata*) have served as a model organism for the study of male coloration, courtship displays, female mate-choice, and visual-pigment evolution [1-8]. Within these, and in other aspects of guppy life-history, a large number of sex- and population-specific morphological and behavioral characteristics have been described [3,9-14], with the most salient of this variation being guppies' pronounced sexual dimorphism. For example, the Cumaná guppy studied here, from a human perspective, exhibits polymorphic black, red, orange, yellow, and iridescent color patterns in males, while females are green, yellow, and iridescent. In areas of low-predation, such as that of Cumaná guppies, male coloration increases and serves as an indicator of fitness to females, while under high predation pressure this coloration is subdued [15,16]. Thus, conspicuous male coloration is a trade-off as it attracts both females and predators [16]. These observations suggest that color vision, at least in females, plays an important role in mate choice and, therefore, in the evolution of variation in male coloration.

Vision is mediated by membrane-bound photoreceptors encoded by opsin genes. The opsin family has been broadly sorted into three subfamilies: photoisomerases, rhabdomeric opsins (r-opsins), and ciliary opsins (c-opsins) (reviewed by Lamb et al. [17,18]). Although r-opsins are used for vision in protostomes the term 'visual opsin' has been used to describe the c-opsins expressed in vertebrate rods and cones. Visual opsins have been categorized into five spectral classes: LWS, SWS1, SWS2, RH2, and RH1 [19]. The first four of these opsin classes facilitate cone-based photopic (bright light) vision and color perception, while RH1, the most recently evolved of the five spectral classes [20,21], facilitates rod-based scotopic (low light) vision. Visual-opsin gene loss and duplication and divergence have generated extensive repertoire variation among vertebrates that, in many cases, correlates to spectral environment (see reviews [22,23]).

Remarkably, the Cumaná guppy genome encodes at least 11 visual opsins. This repertoire includes one *RH1 *gene, three short wavelength-sensitive genes (*SWS1*, *SWS2A*, and *SWS2B*), two middle wavelength-sensitive genes (*RH2-1 *and *RH2-2*), and four long wavelength-sensitive (LWS) genes: *A180*, *P180*, *S180*, and *S180r*. The eleventh gene is a hybrid locus (*A180 *with a *P180 *3' end) that has not been detected in other guppy populations [6-8,24]. The observation that amino acid substitutions have occurred at key sites, even among recent duplicates (e.g., *A180 *and *S180*) [8] suggests that much, if not all, of this diversity is a product of selection.

Long before the guppy opsin gene repertoire was characterized, microspectrophotometry (MSP) data indicated that guppies had several distinct classes of retinal photoreceptor cells (hereafter referred to as, 'photoreceptors'). These include ultraviolet (UV), short- and middle-wavelength sensitive cones with maximal absorption (λ_max_) at 359/389, 410, and 465 nm, respectively, as well as one or more of three LWS cones with λ_max _values clustering at 533, 543, and 572 nm. Rod λ_max _was also characterized at about 503 nm [25-27]. Collectively, these findings implicate photoreceptor-level expression of at least six of the 11 visual-opsin genes found in guppies, though it has not been possible, until now, to associate each λ_max _to a specific opsin and photoreceptor morphology (or 'type').

In several fish species a large visual-opsin gene repertoire appears to play an important role in the tuning of vision for particular environments (see reviews [28,29]). While these examples of vision tuning deal with wavelength sensitivity, evidence of increased color discrimination by means of an expanded opsin gene repertoire has recently been shown in non-teleost species. Transgenic mice expressing three instead of two LWS opsins exhibited enhanced discrimination of red and orange wavelengths of light [30]. Similarly, Mancuso et al. [31] demonstrated that it was possible to treat adult squirrel monkey (*Saimiri sciureus*) congenital red-green color blindness using *LWS*-recombinant adeno-associated virus (rAAV) gene therapy. The intriguing implication of these studies is that retinal circuitry and higher order neuronal processes are either sufficient or adapt to mediate this augmentation to congenital vision. Thus, the potential for guppies to have increased color sensitivity and/or discrimination seemingly hinges on if, and to what extent, these 'extra opsins' are expressed in the retina.

Although all 10 non-hybrid visual opsins are expressed at the mRNA level in Cumaná guppy eyes [8,24], little is known about the relative abundance of each opsin. Ward et al. [8] indicated that *LWS *expression is predominated (>97%) by *A180 *transcripts; however, only three adult fish were surveyed. Here we used reverse transcription quantitative PCR (RT-qPCR) to characterize expression levels of all non-hybrid visual opsins. We surveyed males, females, and juveniles in order to test the hypothesis that sexual selection influences color vision in guppies. As adults, males exhibit elaborate red, orange, and yellow colorations that strongly influence female mate choice [2-4,8,32-34]. Since embryos and juveniles do not show these characteristic colors, and by definition are not sexually active, we predicted that if opsin gene expression plays a role in color-based sexual selection, then *LWS *upregulation will coincide with sexual maturity. Finally, the recent discovery that *SWS2 *and *LWS *occur in a tandem array in Cumaná guppies [35] allowed us to test the hypothesis that distance from a candidate LCR influences opsin expression in guppies, as is the case for human *LWS/MWS *[36,37] and zebrafish (*Danio rerio*) *RH2*s [38].

## Methods

### Animal care and treatment

Care and treatment of guppies were in compliance with the Canadian Council of Animal Care and approved by the University of Victoria Animal Care Committee. Guppies from our inbred lab population (established from specimens collected in Cumaná, Venezuela) were housed in 15 gallon aquaria (unenriched environment) under a synthetic 14:10 hour light-dark cycle with instantaneous transition between photoperiods. Illumination was provided by fluorescent bulbs (General Electric F32T8SP/65K). Relative irradiance of the spectral environment was measured by a USB2000 spectrophotometer (Ocean Optics, Inc.) at the air to water boundary (see Additional file 1). Aquaria were maintained at 24 ± 1°C on a fresh water flow-through system at the University of Victoria Aquatic Research Facility. Fish were fed brine shrimp (*Artemia salina*) flake (Aquatic Ecosystems^®^) daily between 10:00-11:00 hours of the light phase. The number, sex, and age of guppies at time of sampling are detailed in Table 1.

**Table 1:** Guppy housing and rearing

Guppy (n-value) life cycle stage when sequestered in light-dark cycle	Number of offspring in brood	Age at time of sampling^a^	Total RNA [ng/μL] ^b ^and purity (A_260/280_) from pooled ^c ^left eyes	Total RNA [ng/μL] and purity (A_260/280_) from pooled right eyes
Primary survey				

Gravid female (1)	9 (brood-1)	1-month juveniles ^d^	272.9 (2.08)	554.9 (2.09)
Gravid female (1)	12 (brood-2)	1-month juveniles	396.4 (2.10)	429.2 (2.07)
Gravid female (1)	8	2-month juveniles	429.8 (2.10)	470.5 (2.11)
Males (10)	-	Adult	535.0 (2.07)	672.0 (2.10)
Females (6)	-	Adult	441.6 (2.14)	488.3 (2.10)

Secondary survey				

Males_1 _(5)	-	Adult	544.8 (2.10)	
Males_2 _(5)	-	Adult	555.6 (2.07)	
Males_3 _(5)	-	Adult	250.8 (2.09)	
Males_4 _(5)	-	Adult	463.8 (2.03)	
Females_1 _(5)	-	Adult	803.4 (2.10)	
Females_2 _(5)	-	Adult	776.3 (2.10)	
Females_3 _(5)	-	Adult	158.8 (2.11)	
Females_4 _(5)	-	Adult	753.2 (2.12)	

^a ^Euthanasia, followed by immediate eye excision, RNA extraction, and cDNA synthesis.

^b ^Concentration and purity are mean of duplicate measurements within 5ng/μl of each other.

^c ^Each RNA pool corresponds to 1 of the 18 cDNA samples subsequently generated.

^d ^1 month = 28 days.

Note both 1- and 2-month-old juveniles are generally sexually indistinguishable (i.e., not sexually dimorphic); for brevity, the designations 'male' and 'female' are only assigned to adults in this study. Males and females were exposed to each other (i.e., kept in the same aquaria). Male and female guppy numbers (subscripts 1-4) identify different groups.

### Extraction of total RNA and cDNA synthesis

Following light-dark cycle adaptation period (a minimum of 2 weeks for adults) guppies were euthanized in 1 g/L tricaine methanesulphonate, MS222, (Sigma^®^) at 13:00 hours of the light phase. Sampling time was based on cichlid and zebrafish studies indicating that the highest level of cone opsin expression coincides with the end of the photopic day [39,40]. Eyes from 45 individuals were excised and pooled according to age, sex, and left or right eyes (see primary survey, Table 1), with each pool individually placed into 1.0 mL PureZOL™ (Bio-Rad^®^) containing a 3 mm tungsten carbide bead. Extraction of left and right eyes were alternated among individuals to eliminate RNA degradation bias that might have occurred between specimen death and eye immersion in PureZOL™ (a total of 1 minute for the first eye, and 2 minutes for the second eye). To assess variability between broods of the same age, one-month-old juveniles from two females were sampled (primary survey, Table 1). Four additional groups of five individuals, for each sex, had both eyes pooled to expand the *A180 *and *S180 *expression dataset (see secondary survey, Table 1).

Eyes immersed in PurZOL™ solution (plus carbide bead), were homogenize in a Retsch^® ^MM301 Mixer Mill at 20 Hz for 6 minutes. Total RNA was extracted using the Aurum™ Total RNA Fatty and Fibrous Tissue (BioRad^®^), which included a 25 minute DNase I incubation. Concentration and purity (A_260_/A_280_) of total RNA was measured using a ND-1000 Spectrophotometer (NanoDrop^®^) (Table 1). iScript™ cDNA Synthesis Kits (Bio-Rad^®^), which incorporates random hexameric and oligo-dT primers to yield a cDNA representation of total RNA at time of sampling, were used to generate single-stranded cDNA from 2000 ng total RNA per 40 μL reaction. This yielded total cDNA concentrations of 50 ng/μL for each sample, assuming close to 100% conversion. cDNA samples were diluted 50-fold for subsequent RT-qPCR and 10-fold for subsequent reverse transcription PCR (RT-PCR) assays; all cDNA was stored at -20°C.

### Primer design

Locus-specific primers flanking (or spanning) at least one intron were designed for opsin and reference genes from alignments of fish sequences (Table 2). Primers were synthesized by either Operon^® ^Biotechnologies or Integrated DNA Technologies^® ^and stored in sterile dH_2_O at -20°C (Table 2). RT-PCR, using opsin and reference gene primers, was carried out on 10-fold diluted cDNA samples. In each case a single band of the desired size (Table 2) was detected and gene identity confirmed via sequencing at the Centre for Biomedical Research (CBR) DNA Sequencing Facility at the University of Victoria. Due to the high sequence similarity among *LWS*, primer specificity was confirmed by cross-amplification reactions (see Additional file 2) before commencing RT-qPCR.

**Table 2:** Oligonucleotide primers and sequences

Visual opsin gene^a^, reference gene^b^, or plasmid^c^	Primer Name	Primer Sequence (5'→3')	Amplicon size (bp) and GenBank accession number	Primers designed against GenBank accession number or reference
*LWS A180^a^*	A180SpecExon2	GGGTTTACAACGTCTCCACTC	(1017)	[8]
	A1803'UTR-DS	CAATGCAACTATGTTCATT	HQ260679	DQ168659
*LWS P180^a^*	pExon2	GATGGGTTTACGATGTCGCAACGG	(1020)	[8]
	LWS2 IntRev	CAGTCCCGGCAGTAATAACAAAC	HQ260680	
*LWS S180^a^*	a/sExon2	GATGGGTTTACAACGTCTCCACAC	1032	[8]
	LWS1 IntRev	CATTTGCCATAAAGTTTCCGTTTATC	HQ260681	
*LWS S180r^a^*	S180rEx1-Ex2span-S	GCAATCATACAAGAGATCC	(1082)	[35]
	S180r3'UTR-S	ACACAAAACCTTCACTTTTAAGC	HQ260682	
*RH1^a^*	RH1-F	ATGCATGGCTACTTTGTCC	(538)	DQ912024
	RH1-S-R	GTGTGAAGATGTACCAGGCCACAC	HQ260686	
*RH2-1^a^*	RH21-F	CATGGTGGACCCAATGATCTA	(827)	DQ234859
	RH21-R	AGATAACAGGATTGTAAAGG	HQ391990	
*RH2-2^a^*	RH22-F	CAGATCCATGGCAATTCAAA	(738)	DQ234858
	RH22-R	TAAAGAAAATCCACGCAGCA	HQ260683	
*SWS1^a^*	SWS1-F	TGCAGGCGCTCTTCATGG	(799)	DQ234861
	SWS1-R	ATGAGCGGGTTGTAGAC	HQ260685	
*SWS2B^a^*	SWS2B-F	AGGGAGCCTGGGGCTTTTT	(803)	DQ234860
	SWS2B-R	TGGAGGCTTTTGAGACACAG	HQ391991	
*SWS2A^a^*	SWS2A-F	GGCACTTCCATCAACACC	(480)	FJ711159
	SWS2A-R	GAAGCAGAAAAGGAACATGACG	HQ260684	
COI^b^*	Cyt-c-F	GAGCCCTTAAATGGGAGACC	(445)	ES373762
	Cyt-c-R	GCTGCGAAAGCTTCTCAAAT	HQ391989	
*β-actin ^b^*	β-actin-F	GAAACCGGTTCCCTTAAAGC	(439)	EU143772.1
	β-actin-R	GGGGTGTTGAAGGTCTCAAA	HQ260687+	
*Myosin-HC ^b^*	Myosin-HC-F	ACACCAGCCTTCTGAACACC	(491)	ES371087
	Myosin-HC-R	CTCGGCCTGTCTCTTGTAGG	HQ260688	
pGEM-T ^*c*^	SP6	TATTTAGGTGACACTATAG	MCS (141) + Insert	Promega^®^
pGEM-T ^*c*^	T7	TAATACGACTCACTATAGGG		

*cytochrome c oxidase subunit 1.

### Development of plasmid standards

Preliminary qPCR assays, using the methods described below, revealed amplification efficiencies (Eff = 10^(-1/slope) ^- 1) of several opsins to be significantly different from one another (SD >13%) (see Additional file 3), indicating that relative quantification among opsins would not be acceptable. Therefore, Effs were determined by standard curves run in parallel with cDNA samples for each gene during RT-qPCR (see below). To make these plasmid standards, RT-PCR products were generated using opsin- and reference-gene specific primers (Table 2), with subsequent amplicons purified using QIAquick^® ^PCR Purification Kit, A-tailed with Native Taq Polymerase (Invitrogen^®^), and cloned using the pGEM^®^-T Easy Vector System II kit (Promega^®^). Verification of clone insert size was determined by plasmid-PCR, using SP6 and T7 multi-cloning site (MCS) primers (Promega^®^) (Table 1), followed by sequencing (CBR or Macrogen, USA). Plasmids (with opsin or reference gene inserts) were serial diluted to 10^7^, 10^6^, 10^5^, 10^4^, and 10^3 ^copies per 16.3 μL reaction for generation of standard curves by RT-qPCR (detailed below).

### Real-time RT-qPCR

Quantification of visual-opsin and reference gene transcript copy number for the 18 guppy-eye cDNA samples (Table 1) was determined by RT-qPCR analysis carried out in a Stratagene^® ^Mx4000^® ^Multiplex Quantitative PCR machine using gene specific primers (Table 2). Each 16.3 μL reaction was run in triplicate and consisted of: 4.8 mM Tris base, 4.4 mM Tris-HCl, 46 mM KCl, 3.68 mM MgCl_2_, 0.009% Tween-20, 0.74% glycerol, 0.0023% SYBR Green I (Invitrogen^®^), 184 μM dNTPs, 920 nM of each primer, 1.0 U Platinum Taq (Invitrogen^®^), 5 μL of 50-fold diluted cDNA, and 3.83 nM ROX Passive Reference Dye (Invitrogen^®^) used to normalize the fluorescent reporter signal between reactions. RT-qPCR conditions consisted of 1 cycle at 95°C (9 minutes); 50 cycles of 95°C (15 seconds), 60°C (30 seconds), 72°C (45 seconds); 1 cycle of 95°C (1 minute); followed by a 41-step melting-curve analysis (initial temperature 55°C, increasing 1°C every 30 seconds). Cycle threshold (Ct) values, based upon the normalized change in fluorescence (dRn), for each locus were compared to a corresponding standard curve run in parallel to determine transcript copy number. These values were then normalized to the transcript copy number (geometric mean) of the three reference genes (Table 3) (see Additional file 4). See Additional file 5 for justification of reference gene use. Dissociation curves (Fluorescence [-Rn'(T)] over T°C) and agarose gel electrophoresis were used to confirm the presence of single amplicons.

**Table 3:** Plasmid standard curve values

Plasmid::gene of interest	Linear Equation	Amplification Efficiency (%)	r-squared values (Rsq)
Primary survey			

*A180*	Y = -4.976*Log(X) + 56.76	58.8	0.979
*P180*	Y = -4.539*Log(X) + 51.69	66.1	0.981
*S180r*	Y = -3.494*Log(X) + 43.42	83.8	0.993
*S180*	Y = -3.998*Log(X) + 51.22	77.9	0.986
*RH2-1*	Y = -4.141*Log(X) + 52.61	74.4	0.995
*RH2-2*	Y = -3.846*Log(X) + 41.51	82.0	0.996
*SWS2A*	Y = -3.881*Log(X) + 43.15	81.0	0.942
*SWS2B*	Y = -4.142*Log(X) + 49.30	74.4	0.993
*SWS1*	Y = -3.378*Log(X) + 38.49	97.7	0.998
*RH1*	Y = -4.036*Log(X) + 39.05	76.9	0.990
*COI*	Y = -3.828*Log(X) + 41.05	82.5	0.991
*B-actin*	Y = -3.402*Log(X) + 38.67	96.8	0.996
*Myosin-HC*	Y = -3.651*Log(X) + 39.80	87.9	0.999

Secondary survey			

*COI*	Y = -4.132*Log(X) + 47.75	74.6	0.957
*B-actin*	Y = -3.861*Log(X) + 42.84	81.6	0.973
*Myosin-HC*	Y = -4.217*Log(X) + 50.74	72.6	0.988
*A180*	Y = -3.774*Log(X) + 43.27	84.1	0.998
*S180*	Y = -4.184*Log(X) + 51.10	73.4	0.984

Data shown are from triplicate RT-qPCR reactions run in parallel with guppy cDNA samples, and were used to determine opsin- or reference-gene transcript copy number.

## Results

**Figure 1:**
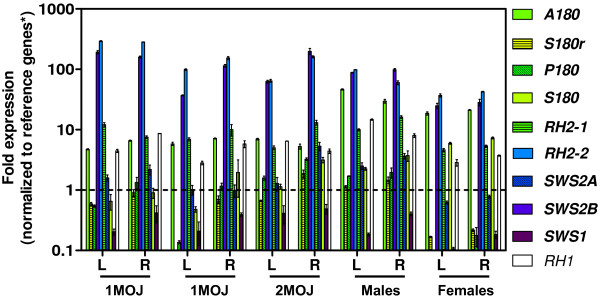
**Fold expression of guppy visual opsin genes in juvenile and adult eyes**. One- and two-month old juvenile (1MOJ and 2MOJ, respectively) and adult (male and female) cDNA samples are derived from left (L) and right (R) eye mRNA pools isolated from 6-12 individuals for each age or sex (see Table 1). Opsin transcript copy number was normalized to the transcript copy number (geometric mean, represented by dashed line) of three reference genes: *COI*, *β-actin*, and *Myosin-HC *(asterisk). Error bars (±S.E.M. of triplicate reactions) are shown for all samples. Colors represent the λ_max _of each opsin (based on Wavelength to RGB v.1.0 software), excluding RH1.

**Figure 2:**
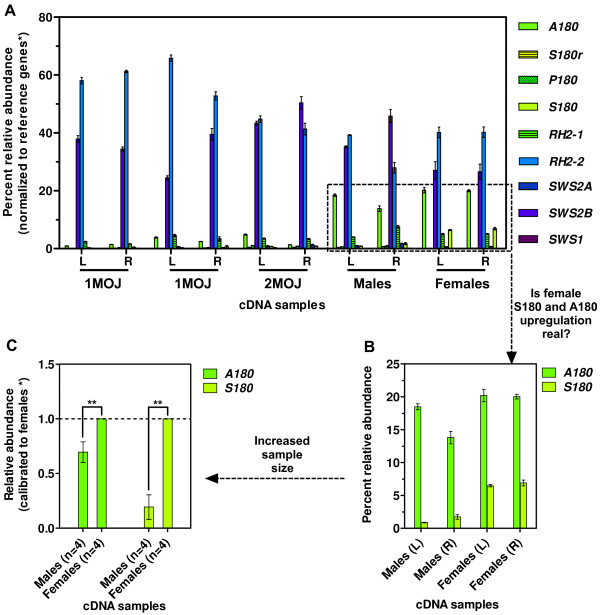
**Relative abundance of putative cone opsin transcripts for**: (**A**) one- and two-month old juvenile (1MOJ and 2MOJ, respectively) and adult (male and female) cDNA samples derived from left (L) and right (R) eye mRNA pools isolated from 6-12 individuals for each age or sex (see primary survey, Table 1). Error bars (±S.E.M. of triplicate reactions) are shown for all samples. (**B**) Inset magnifying *A180 *and *S180 *transcript relative abundance levels from panel A. (**C**) cDNA from four groups (n = 4) of five individuals (left and right eyes pooled) for each sex (see secondary survey, Table 1) used to determine if the slight *A180 *and large *S180 *upregulation in females observed in panel B is real. A statistically significant, p < 0.005 (asterisks), difference was observed between males and females for both *A180 *and *S180 *expression (based on independent student's unpaired *t*-tests of data before calibration to female expression). Error bars (±S.E.M. of four biological replicates) are shown. Note 'percent' relative abundance could not be calculated as only two of the nine 'cone' opsin genes were assayed. (**A-C**) Opsin transcript copy number was normalized to the transcript copy number (geometric mean) of three reference genes (asterisk): *COI*, *β-actin*, and *Myosin-HC*. Colors represent the λ_max _of each opsin (based on Wavelength to RGB v.1.0 software).

Guppies express all 10 non-hybrid visual opsins in the eye at varying levels depending on age and sex (Figure 1). In juveniles, two genes (*SWS2B *and *RH2-2*) accounted for >85% of visual opsin transcripts in the eye, excluding *RH1 *(Figure 2A). In adults, the relative abundance (RA) of *SWS2B *and *RH2-2 *transcripts decreased to about 65%, apparently making room for the increase in *A180 *transcripts from approximately 3% to 20%, while the juvenile-to-female transition showed *S180 *upregulation from about 1.5% to 7% (Figure 2A-B). In all samples, *A180 *was the predominant *LWS *expressed (Figure 2A). Data from our secondary survey showed a statistically significant (p < 0.004) increase in female *S180 *expression and a small but statistically significant (p < 0.005) increase in female *A180 *expression relative to males (based on independent student's unpaired *t*-tests for each gene; statistical significance was set at: p < 0.05) (Figure 2C). We also detected a small amount of variation (0.32%, p < 0.0001) between left and right eye expression (based on two-way ANOVA of all left and right eye values); however, no significant difference (p = 0.6136) was seen between left and right eye expression overall (student's unpaired *t*-test; statistical significance was set at: p < 0.05). Mean expression across cDNA samples for each opsin gene within the *SWS2-LWS *cluster was higher for genes closest to the candidate LCR, while *S180r*, unlinked to the other *LWS *[41], showed the least expression.

## Discussion

The recent discovery that Cumaná guppies (*P. reticulata*) express 10 of their 11 visual-opsin genes in adult eyes [6,8,24], suggested that this species possesses enhanced wavelength sensitivity and/or discrimination. Characterization of this repertoire was particularly exciting given the wealth of data on color-based sexual selection in this species. However, RT-qPCR data reported here indicates that the guppy eye might not be the irradiance detector we had initially envisioned.

### Two to four visual opsins predominate in guppy eyes

Our RT-qPCR analysis of Cumaná guppy eyes revealed that all 10 non-hybrid visual-opsin genes are expressed in juveniles and adults (males and females) (Figure 1). Curiously, only two opsin genes in juveniles (*SWS2B *and *RH2-2*), three in males (*SWS2B*, *RH2-2*, and *A180*), and four in females (*SWS2B*, *RH2-2*, *A180*, and *S180*), produced the majority of visual opsin transcripts in the eye, excluding *RH1 *(Figure 2A). At first, these surprising results suggest that juveniles, males, and females are at least dichromatic, trichromatic, and tetrachromatic, respectively. However, any extrapolations from opsin expression to color vision require data on the location of opsins in the guppy retina, not just their quantity. To shed light on this, we compared our RT-qPCR data to MSP data on guppy cone cells. This allowed us to assign visual opsins to λ_max _peaks. As λ_max _peaks are known for specific cone types, and for specific regions of the retina, we were able to assign opsins to different cone types and areas of the retina.

### Guppy visual-opsin expression in rods and cones

**Figure 3:**
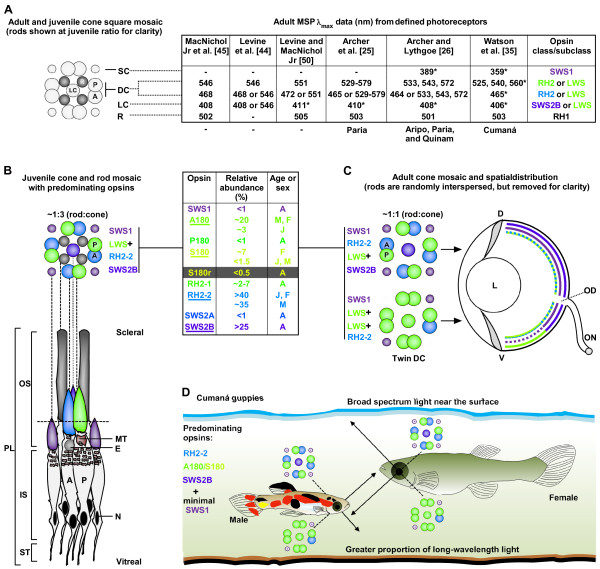
**Guppy vision: cone mosaic and visual opsin expression pattern**. (**A**) A square cone-mosaic is present in adults and juveniles. Adult MSP derived λ_max _values from multiple studies and populations (listed below) are shown for each photoreceptor type: short single cone, SC; accessory, A, and principle, P, double cone, DC; long single cone, LC; rod, R. Unspecified cones (asterisk) are grouped with like values. Colors represent the λ_max _of each cone opsin (based on Wavelength to RGB v.1.0 software). Not reported (dash). (**B**) Photoreceptor layer (PL) (below) with tangential cross-section (above) showing juvenile rod and cone mosaic with predominating opsins (based on data from panel A and our expression data). Table summarizing our RT-qPCR expression data for the nine putative cone opsins in the eyes of: juveniles, J; females, F; males, M; all ages and sexes tested, A. Predominantly expressed opsins (underlined) constitute >95% of all visual opsin transcripts (excluding *RH1*) in Cumaná guppy eyes. Among populations, differential LWS or RH2-1 expression likely occurs (LWS+). Grey background added for clarity. Additional abbreviations: outer segments, OS, are proportional in length and diameter (adult data); inner segment, IS; synaptic terminal, ST; ellipsosome, E; mitochondria, M; nucleus, N. **(C**) Adult cone-mosaic showing differential DC and LC expression (unknown in juveniles). Median dorsal-ventral section of an adult guppy eye showing cone spatial distribution based on MSP and morphological studies. Decrease in quantity of cone class (or dotted lines) do not reflect exact ratios; lens, L; dorsal, D; ventral, V; optic nerve, ON; optic disc, OD, is devoid of photoreceptors. (**D**) Cumaná guppy dorsal-ventral retinal tuning to environmental spectra; style adapted from reference [66]. This figure is a composite, based on our RT-qPCR data and references [25,26,28,44-47,50,57,66,89-95].

In this section, we first explore previous opsin-to-λ_max _assignments and then show how our RT-qPCR data fills the gaps in these assignments. MSP studies on adult guppy retinas demonstrated the presence of: rods with a λ_max _at about 503 nm, ultraviolet-sensitive (359/389 nm) cones, violet-sensitive (408 nm) cones, blue-sensitive (465 nm) cones, and a series of green- to yellow-sensitive cones with λ_max _values clustering at 533, 543, and 572 nm [25-27]. Inter-specific opsin comparison indicated that these photoreceptors express RH1, SWS1, SWS2B, RH2, and both RH2 and LWS opsins, respectively (Figure 3A) [28]. Hofmann and Carleton [28] attributed the 408 nm peak to SWS2B and not to SWS2A, which is consistent with SWS2B generally exhibiting a lower λ_max _than SWS2A in teleosts (reviewed by Bowmaker [42,43]). However, it was unclear which RH2 (RH2-1 or RH2-2) was responsible for the 465 or 533 nm peaks (discussed below). Now, with our RT-qPCR data, this assignment ambiguity can be clarified, finally enabling visual opsins to be assigned to photoreceptor types.

#### SWS2 and RH2 assignments

Our expression data led us to assign the 408 and 465 nm peaks to SWS2B and RH2-2, respectively. This was due to *SWS2B *and *RH2-2 *transcripts exhibiting a combined RA of >65% among all guppies assayed, whereas *SWS2A *and *RH2-1 *transcripts showed ~5% RA (Figure 3B-box). It is, therefore, likely (assuming opsin expression is proportional to cone cell number) that the above MSP studies detected more of the highly expressed pigments than their less expressed counterparts. These data, combined with the observation that MSP consistently found 464-472 nm peaks for accessory double-cones (DCs) in multiple populations (Figure 3A), led us to attribute the majority of *RH2 *and *SWS2 *expression in juvenile and adult guppy eyes to RH2-2 in accessory DCs and SWS2B in dorsal long single-cones (LCs) (Figure 3B-C). This conclusion, as well as others made below, is contingent on opsin-class expression in photoreceptor types being similar between our Venezuelan population (Cumaná) and that of the above MSP studies done on Trinidadian populations (Aripo, Paria, and Quinam). This is probable given that the λ_max _values for photoreceptors types are consistent among all populations sampled to date, with variation limited to the long wavelength-sensitive region (Figure 3A).

At this point, it is important to note that, although dorsal-ventral differences in opsin expression have not been investigated in Cumaná guppies, dorsal-ventral differences in cone pigment distribution (Figure 3C) were found in another population via MSP [44]. Furthermore, MSP data showing that accessory DCs invariably contain 468 nm pigments (Figure 3A) [45], combined with morphological studies (reported and reviewed by Kunz and Wise [46]) showing that the ventral retina is largely devoid of ellipsosomes (present only in accessory DCs [46,47]), supports the above MSP finding and our proposal that Cumaná guppies exhibit similar dorsal-ventral expression. Finally, the spectral selective-pressures thought to be responsible for this pattern (discussed later) are present in Cumaná guppy habitats.

#### LWS assignments

Variation in λ_max _of LWS cones is present among adult guppy individuals in both Trinidadian and Venezuelan populations. For three Trinidadian populations, one or more peak absorption (clustered around 533, 543, or 572 nm) is exhibited (Figure 3A) [26]. However, as these data represent 15 individuals from each of the three populations (Aripo, Paria, and Quinam) grouped together during analysis, it is unclear if variation in λ_max _is attributed to individual and/or population-level differences. Although, individual variation was, indeed, observed for the Paria population in an earlier study [25]. Similarly, for Venezuelan guppies, recent MSP data indicate that individuals of at least one population (Cumaná) also contain one or more of three LWS cones. These cones have λ_max _values of about 525, 540, and 560 nm, with four out of the seven individuals assayed containing only 540 nm cones [35]. Given this, and that we report data from only our highly inbred Cumaná lab-population, it stands to reason that we should see limited variability in LWS opsin expression - a hypothesis that is supported by *A180 *transcripts clearly predominating in our lab population (Figure 2A), as was indicated in preliminary work [8].

Previously, guppy A180-to-λ_max _assignments have been based on values from other species [48] and on the -7 nm shift attributed to the S180A substitution [49]. These data led Ward et al. [8] to assign A180's λ_max _to 553 nm. However, with both *A180 *transcription and 540 nm LWS cones predominating in Cumaná guppies, we can now attribute these cones to A180 expression. Further support for this assignment comes from a relative of the guppy, the green swordtail (*Xiphophorus helleri*), that expresses P180, S180, and S180r, but lacks *A180 *[41]. The fact that this species does not exhibit an absorption peak near 540 nm [41] supports our assignment of A180 being responsible for the 540/543 nm peak found in guppies. Within a cone mosaic unit, LWS ventral twin-DCs and ventral LCs have been shown to have the same λ_max _values, indicating expression of the same pigment(s) (Figure 3C) [44,50,51]. In light of these data, the principle member of DCs, LWS ventral twin-DCs (the predominant type of DC in the ventral retina of guppies [44,50,51]), and ventral LCs, all appear to express A180 in Cumaná guppies (Figure 3B-C).

MSP showed no significant difference between male and female λ_max _frequencies for LWS cones among guppies from the three different Trinidadian populations mentioned above [26]. Although our Venezuelan population showed a significant difference between male and female *A180 *expression, ~15% versus 20% RA, respectively (Figure 2A-C), this difference might not have been recognized by MSP or may not be present in Trinidadian populations. We also detected a significant increase in female *S180 *expression in our population (Figure 2C). Trinidadian populations exhibit relatively few 560 nm peaks [26], which we assign to S180 (detailed below), indicating that S180 expression appears to differ among populations.

S180 and S180r - the most red-shifted LWS opsins in guppies - were once both predicted to have λ_max _values of approximately 560 nm [7,8]. However, the spectral-tuning effects of the amino acid differences between these two opsins have not been determined [7]. Since female Cumaná guppies express 10 times more *S180 *transcripts than that of *S180r *(Figure 3B-box), and that 560 nm cones are the only cones more red-shifted than A180 cones in this population [35], we attribute these 560 nm cones to S180 expression. This assignment is consistent with the two S180 (SHYTA five-site haplotype) opsins found in medaka (*Oryzias latipes*) having a λ_max _of 561 and 562 nm [52].

Trinidadian guppy populations exhibit LWS cones with λ_max _values at about 572 nm [26]. Given *S180r*'s low transcription and lack of 572 nm cones in our Venezuelan guppy population, as well as S180r's amino acid differences from S180, we attribute the 572 nm cones in Trinidadian populations to S180r expression. This assignment is consistent with at least one of bluefin killifish's (*Lucania goodei*) two LWS opsins being S180r [8], as both LWS-A and LWS-B seem to absorb at 573 nm [53]. Green swordtail also exhibits a similar peak (568 nm), though this is thought to be due to S180 subtypes (S180-1 and/or S180-2) [41]; however, since quantitative expression data is currently unavailable for this species, S180r can not be ruled out.

The above opsin-to-λ_max _assignments for A180, S180, and S180r are also consistent with the predicted λ_max _of P180 being the most blue-shifted LWS opsin at 512 nm (531 nm minus the 19 nm blue-shift of the S180P substitution [8,54]). Since rods are the only retinal photoreceptor near P180's predicted λ_max _(512 nm) for both Trinidadian and Venezuelan guppy populations (Figure 3A), and that we detected <1% RA for P180, it seems that if visual relevancy is exhibited at this expression level in Cumaná guppies, then it may be due to a small number of P180 cones missed by MSP scans or, more likely, coexpression with another opsin that may serve to broaden the spectral absorption range of the cell.

Given our P180, A180, S180, and S180r λ_max _assignments of 512, 540, 560, and 572 nm, respectively, which LWS opsin is responsible for the ~525-533 nm peak found in multiple guppy populations [25,26]? Hofmann and Carleton [28] dissented from the previously held notion that the ~525 nm peak was attributed to a LWS opsin. Instead, they assigned the ~525 nm peak to the RH2 opsin class, which is consistent with other teleost RH2s absorbing in this range [52,53,55,56]. However, it has not yet been discerned whether this peak is due to RH2-1 or RH2-2. Given RH2-1's low expression, relative to RH2-2 (~5% versus ~40% RA, respectively), and the rarity of 525 nm cones among Cumaná guppies [35], it appears that RH2-1 is a strong candidate for explaining the 525-533 nm peaks found in principle DCs among various populations. This assignment is also consistent with RH2-1 absorbing at 537 nm in bluefin killifish [53].

#### SWS1 assignments

Archer and Lythgoe [26] were the first to detect the presence of UV-sensitive single cones with a λ_max _at 389 nm in guppies. Endler et al. [27] later reported (based on a V.N. Rush personal communication) cones with a λ_max _at 359 nm, while recent work by Watson et al. [35] showed identical results. In retrospect, it is understandable that Archer and colleague's early MSP studies [25,26], as well as others preceding it (reviewed in Figure 3A), might have had difficulties detecting UV-sensitive cones due to the scanning range of their measuring beam (~370-730 nm). This appears to account for the lack of short corner-cones (SCs) described in previous MSP studies (Figure 3A). Indeed, ultra-structure studies on adult guppy retinas have shown SCs across the whole retina (Figure 3C), excluding a thin peripheral zone and most of the ventral retina (reviewed by Kunz [57]), which in teleosts generally contain SWS1 [58]. Based on these data, we assign the 359 nm peak to SWS1 expression in SCs, which is consistent with SWS1-to-λ_max _assignments in other teleosts (reviewed by Hofmann and Carleton [28]).

With the above assignment, it is curious that our *SWS1 *expression data among all guppies examined is relatively low (<1% RA) given the apparent quantity of SCs in the retina (Figure 3B-C). This observation may be explained, in part, by ultra-structural analysis that showed a decrease in ventral SCs relative to that of the dorsal retina (Figure 3C) (reviewed by Kunz [57]). Furthermore, the spectral conditions (see Additional file 1) of our population and/or age at sampling might have contributed to these low expression levels. Such minimal SWS1 expression is not unique, as comparable levels are seen in zebrafish juveniles and adults [59], as well as adult medaka [52], while rainbow trout (*Oncorhynchus mykiss*) experience more than a 20-fold decrease in *SWS1 *expression during development from parr to smolt [60].

#### RH1 assignments

*RH1 *expression, located in rods (λ_max _≈ 503 nm) [35], fluctuated among guppy ages without a clear pattern emerging, with overall expression being relatively low compared to cone opsins (Figure 1). This was not expected since adult guppies have an increased number of rods relative to juveniles (Figure 3B-C), which should be reflected by *RH1 *upregulation in adults. Similar to the *SWS1 *expression pattern discussed above, the spectral environment of our population might have affected *RH1 *expression. Although the guppies used in this study were on a synthetic light-dark cycle, the lab population from which they were originally sampled experienced 24-hour artificial light conditions (accompanied by seasonal ambient lighting) since it was established six years prior to the present study. Additionally, guppy rods are known to move sclerally under photopic conditions [57]. This movement increases absorption and recycling of the rod disks by the adjacent retinal pigment epithelium (RPE) [57,61]. Furthermore, in the cichlid, *Haplochromis burtoni*, *RH1 *expression peaks near the beginning of the light cycle [62], while cone opsins are maximally expressed towards the end of the light cycle [39]. With these factors in mind, it appears that low *RH1 *expression in guppies reflects both sampling time and artificial light conditions.

#### Opsin spatial expression

Guppy cone cells are organized into a square mosaic across the retina (reviewed by Kunz [57]). Thus, dorsal- and ventral-mediated vision is probably comparable to central vision with respect to visual acuity; putting equal importance on dorsal-ventral differences in opsin expression with respect to spectral sensitivity and/or discrimination. MSP data indicate that the majority of twin DCs and LCs in the ventral retina of guppies express LWS pigments (Figure 3C). In other fish, dorsal-ventral differences in expression are also seen. Zebrafish, for example, express shorter wavelength-sensitive RH2-1, RH2-2, and LWS-2 in the dorsal retina, while expressing their longer wavelength-sensitive counterparts (RH2-3, RH2-4, and LWS-1) in the ventral retina [38,63]. This expression of longer wavelength-sensitive opsins in the ventral retina of diurnal shallow-water species appears to be, at least partially, a result of the transmission properties of the water [50].

Guppies are a tropical, freshwater, surface dwelling species native to Trinidad and Venezuela [3,26,50], and are found in clear to tea colored water [64,65]. The majority of these freshwater environments contain dissolved/suspended organic material that tends to absorb short-wavelength light, resulting in increased transmission of long-wavelengths through the water column (reviewed by Levine and MacNichol [50]). This appears to be reflected in the LWS-dominant ventral retina of the guppy, which at increasing depths seems tuned to the most abundant downwelling light (long-wavelengths), thereby maximizing contrast of objects above (e.g., food, predators, and conspecifics) (Figure 3D) [50,66]. However, this adaptation may be at the cost of color discrimination, due to a single cone class (LWS) predominating in this region [50,66]. Conversely, the dorsal-central retina seems well suited for color discrimination given the presence of at least three different cones pigments [50,66], where we propose that spectrally diverse opsins (SWS2B, RH2-2, and A180, as well as S180 in females, for Cumaná guppies) predominate (Figure 3D). This opsin expression pattern would be fitting since males perform courtship displays most often in front and sometimes slightly below females [67,68].

#### Opsin coexpression

Complicating the interpretation of opsin expression and MSP data is the potential for coexpression of two or more visual opsins in a cone cell. This might explain the low expression of some of the opsin genes detected in our guppy population. Coexpression may serve to expand the absorption range of guppy cones, as this appears to be the case for the Siberian hamster [69]. Recently, rainbow trout have been shown to coexpress RH2 duplicates during the parr-to-smolt transition, yielding a cone with a broader absorption range than other classes [70]. However, coexpression is likely to diminish photoreceptor sensitivity to a specific wavelength if total opsin expression in that cell is constant.

#### Individual opsin expression

Low-abundance opsins may play a key role in guppy vision even if their transcript levels are a consequence of expression in relatively few cones cells. For example, humans have approximately 92 million rods and about 4.6 million cones per eye [71]. Thus, about five percent of photoreceptors are responsible for generating color vision, while only 5-10 percent of this population is comprised of SWS cones (reviewed by Calkins [72]). Despite this relatively small quantity of SWS cones, humans can readily detect short wavelengths of light, and when *SWS *is not functional or absent (i.e., tritanopia, or 'blue-yellow color blindness') the effect on color discrimination is often dramatic (reviewed by Bowmaker [73,74]). With this in mind, one should not discount the potential role of even a small population of guppy cone cells. Alternatively, it is possible that one or more of the opsin genes with low-abundance transcription are upregulated at a time not represented in this study (e.g., embryonic development).

### *LWS *upregulation in adult guppies

In the previous section we formed a model of opsin expression in the guppy cone mosaic that is consistent with the selective pressures of environmental spectra and sexual selection. With this in mind, we now seek to identify the evolutionary forces that have led to this species' *LWS *expression pattern.

An important finding of this study is the marked upregulation of *A180 *from near 3% RA in juveniles to about 20% RA in adults (Figure 2A). These data may have far-reaching implications, as it supports our hypothesis that if opsin gene duplication and divergence plays a role in sexual selection then we expect to see *LWS *upregulation coinciding with sexual maturity (i.e., adulthood). This result thereby implicates A180 in conferring adult guppies with increased spectral sensitivity and/or discrimination. However, the selective pressures that may have led to, and/or maintained, this upregulation appear to differ between sexes.

For males, we propose that *A180 *upregulation enhances detection of foods with greater quantities of carotenoids. This ability would increase conspicuousness (i.e., spot coloration), as guppies do not synthesize carotenoid pigments *de novo*; instead, the acquisition and subsequent modification of such pigments starts with ingestion of carotenoids found in the fruit and algal component of their diet [75-77]. Being able to better detect food would increase overall fitness, while acquisition of foods with greater amounts of carotenoids would increase the color saturation of orange spots [75,76,78], which is an indicator of foraging ability [76,79] and immunocompetence [80,81]. In turn, reproductive fitness levels would rise since females from many populations show a preference for males with greater orange spot area and color saturation [4,64,75,76]. Together, these factors are strong candidates for selective forces driving *A180 *upregulation in male guppies.

For females, we propose that increased A180 expression would allow for enhanced spectral discrimination of male spot coloration - long known to be an integral trait assessed by females in a prospective mate [2-4,32] - or provide improved detection and evaluation of male sigmoid displays. The latter of which was proposed by Ward et al. [8] regarding non-specific *LWS *upregulation. Interestingly, the juvenile-to-female transition also showed upregulation of *S180 *from about 1.5% to 7% RA (Figure 2A and 2C), which further supports our hypothesis of *LWS *upregulation at sexual maturity, suggesting that increased S180 may also enhance discrimination of male coloration or courtship displays. Furthermore, our secondary survey detected small but statistically significant *A180 *upregulation in females relative to males (Figure 2C), which may also augment spectral discrimination of male coloration. Importantly, these data are consistent with laboratory and wild guppy disk-pecking experiments that showed both sexes are attracted to orange colors, while females exhibited a greater interest for orange and males for yellow [77], which may reflect female upregulation of *S180 *and/or the small upregulation (relative to males) of *A180 *detected in the present study. Additionally, it is possible that females also benefit from consumption of carotenoid-rich food, as guppy egg carotenoid levels positively correlate with dietary intake; although, offspring quality does not appear to be affected [78]. Intriguingly, the only other known taxa with sex-specific differences in opsin gene expression are the lycaenid butterflies, where color-based sexual selection has also been demonstrated [82].

For both male and female guppies, it has been proposed that increased expression of spectrally distinct opsins can aide in motion detection [83], which may heighten male (possibly rival male) detection or predator avoidance. However, if the increased expression and number of LWS opsins seen in adults confers an adaptive advantage with respect to predation, then one would expect upregulation of these genes to be selected for at earlier developmental stages (i.e., juveniles). This indicates that upregulation of *A180 *and *S180 *confers an adaptive advantage to events that occur later on in life, such as rival male detection, food acquisition, or mate choice. It is, therefore, fitting that disk pecking experiments also showed a lack of juvenile preference between orange and red colors, which may reflect an inability to discriminate between the two, while adults were able to make this distinction [77]. This suggests that adults have increased discrimination of long wavelengths of light - an ability usually afforded by additional spectrally-distinct LWS opsins [30,31], which we propose is a function of the *A180 *and *S180 *upregulation only observed in adults. Furthermore, no differences in color preference were detected between guppies from high- versus low-predation populations [77], indicating that color discrimination is not linked to predation.

Our above proposal is also consistent with Rodd et al.'s [77] work, which indicates that female guppies' preference for male orange-coloration has developed as a product of a sensory bias for orange-colored objects, such as carotenoid-rich fruits. Thus, the high expression of *A180 *in males and their ability to discriminate longer wavelengths of light is not surprising, as it probably was beneficial for both sexes to be able to detect fruit before female's preference for male coloration emerged.

### Effects of LCR proximity on opsin gene expression in guppy eyes

**Figure 4:**
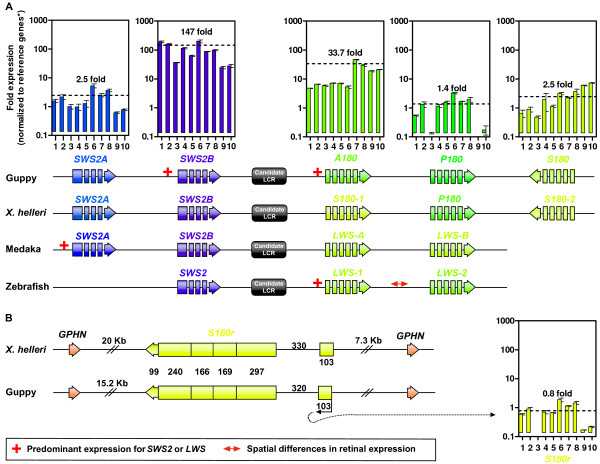
**Ocular expression and genomic architecture of *SWS2 *and *LWS *in guppies and relatives**. (**A**) Histograms of guppy opsin expression (mean = dashed line) from one- and two-month old juvenile and adult cDNA samples (shown in the same order as Figure 2A). Opsin transcript copy number was normalized to the geometric mean of three reference genes (asterisk): *COI*, *β-actin*, and *Myosin-HC*. Error bars (±S.E.M. of triplicate reactions) are shown for all samples. Colors represent the λ_max _of each opsin (based on Wavelength to RGB v.1.0 software). Expression (when known), genomic organization, and λ_max _data are shown for: adult and juvenile guppies (see text for opsin-to-λ_max _assignments) [8,28], adult green swordtail, *X. helleri*, (note *SWS2A *and *P180 *λ_max _assignments are uncertain [41]), adult medaka [52], and adult and juvenile zebrafish [59]. Intergenic, candidate LCR, and exon/intron lengths are not to scale. (**B**) Location of *S180r *in intron XI of *gephyrin *(*GPHN*) in *X. helleri *[41] and guppy [35]. Guppy *S180r *expression (right) adheres to the parameters described for panel A. *S180r *exon-intron lengths are to scale and are represented in number of base-pairs unless otherwise indicated (i.e., kilo-bases (Kb) used for large intronic sequences); data are from: references [8,35,41] and the present study.

Recently, the genomic organization of *SWS2 *and *LWS *were reported for guppies [35] and a close relative, the green swordtail (*X. helleri*) [41]. This organization consists of a tandem array where *SWS2 *is located upstream of a candidate LCR with *LWS *following downstream (Figure 4A). This pattern is also found in medaka, zebrafish, stickleback, fugu (*Takifugu rubripes*), Tetraodon (*Tetraodon nigroviridis*), and in monotremes (e.g., platypus, *Ornithorhynchus anatinus*, and echidna, *Tachyglossus aculeatus*) [41,84]. Of these species, those with more than two opsins in the array are shown in Figure 4A.

For guppies, Watson et al. [41] hypothesized that if *A180 *is located closest to the putative LCR then this may explain its relatively high expression among *LWS*, as distance from a LCR has been shown to negatively correlate with opsin expression (reviewed by Watson et al. [41]). Now that the genomic location of these genes are known for guppy, we expand on this hypothesis in that both *SWS2B *and *A180 *should be the most highly expressed genes in the *SWS2-LWS *gene cluster if distance to the LCR negatively correlates with expression. Our results indicate that this might be the case, with *SWS2B *and *A180 *exhibiting predominant expression in this gene cluster (Figure 4A). Zebrafish also exhibit highest expression of their most proximal *LWS *(*LWS-1*) [59], while both *LWS-1 *and *LWS-2 *exhibit different spatial expression [38,63]. However, medaka deviate from this pattern with *SWS2A *expression exceeding *SWS2B *(Figure 4A), while expression data for its two *LWS *(*A *and *B*) are unknown since primers that can distinguish between the two were not used [52]. Currently, quantitative expression data for *X. helleri *opsins are also unknown.

If the above upregulation is solely a consequence of proximity to the LCR, then A180 levels should be consistent among populations since this genomic architecture would not likely vary. However, it is clear that among teleosts, locus distance to a LCR does not always negatively correlate with expression, implying that other *cis*- or *trans*-acting factors also regulate expression in this gene cluster, which may explain the differences in LWS-cone maximal absorption among populations and individuals.

### *S180r *genomic location and expression

Both guppies and *X. helleri *possess a fourth *LWS *(*S180r*) that lacks introns II-V and is located in intron XI of *gephyrin *(*GPHN*) (Figure 4B), which suggests that *S180r *is a result of retrotransposition [8,35,41]. This is consistent with retrogenes typically inserting into areas of the genome unlinked to the progenitor gene (reviewed by Kaessmann et al. [85]). Orthologs of *S180r *have also recently been identified in the one-sided livebearer (*Jenynsia onca*) and the four-eyed fish (*Anableps anableps*) [24,86]. Furthermore, exon/intron I structure and length are highly conserved between guppy *S180r *and its progenitor gene, *S180*, as is the case in *X. helleri *(Figure 4B), indicating that *S180r *did not obtain its single intron post retrotransposition.

While the intron that survived retroduplication might bear enhancers that promote expression in the retina, it is also possible that the *S180r *transcripts we detected in guppy eyes were driven by *GPHN *regulatory elements. However, since *S180r *is in the reverse orientation to *GPHN *(Figure 4B), *GPHN *pre-mRNA (i.e., before intron splicing) could not additionally function as the coding strand for the S180r protein; although, the *S180r *non-coding region could be converted into cDNA and amplified by RT-qPCR, yielding the appearance of low-level coding-transcripts where there may be none. Nonetheless, transcription of *S180r *(i.e., yielding coding strand mRNA) in the eye may occur via other mechanisms (Figure 4B). One such mechanism stems from GPHN being present at inhibitory γ-aminobutyric acid (GABA) synapses where it aids in the translocation and stabilization of GABA receptors to the postsynaptic membrane (reviewed by Sassoe-Pagnetto [87]). Thus, it would be highly expressed in guppy eyes since there are a myriad of GABAergic synapses present in the vertebrate retina [88]. *GPHN*'s high expression would probably be accompanied by an increasingly open chromatin structure where retrogenes are more accessible to transcriptional machinery [85], while neighboring *GPHN *enhancers may also facilitate *S180r *transcription.

## Conclusion

Our RT-qPCR analysis of visual-opsin gene expression in juvenile and adult guppies has enabled us to propose the first opsin-to-λ_max _assignments for all photoreceptors in the cone mosaic. Where SWS1 (λ_max _≈ 359 nm) is expressed in SCs, SWS2B (λ_max _≈ 408 nm) is expressed in LCs, RH2-2 (λ_max _≈ 465 nm) is expressed in the accessory member of DCs, and LWS opsins (A180, λ_max _≈ 540 nm, predominating in Cumaná guppies) are expressed in the principle member of DCs, LWS ventral twin-DCs, and ventral LCs. Variation in λ_max _of LWS cones among guppies appears to be a result of individual and/or population level differences, which likely explains the low-abundance *LWS *transcripts detected in the present study. Although our results indicate that Cumaná guppy juveniles, males, and females are at least di-, tri-, and tetra-chromatic, respectively, it is important to keep in mind that opsin coexpression and differential spatial-temporal expression may enable low-abundance opsins to be visually relevant. *in situ *hybridization experiments will be carried out to test this hypothesis.

Marked *LWS *(*A180 *and to lesser extent *S180*) upregulation in adults coincides with males donning orange and red spots, whose area and color saturation positively correlate with reproductive fitness and carotenoid-rich food ingestion. Given this, and that behavioral studies suggest that adults may have greater discriminatory abilities at longer wavelengths light than juveniles, *LWS *upregulation in adults is implicated in enhancing spectral discrimination of male coloration and carotenoid-rich foods, with the former seeming to have arisen from a sensory bias of the latter.

In the *SWS2-LWS *gene cluster, genes closest to the candidate LCR, *SWS2B *and *A180*, exhibited the highest expression. However, locus distance to a LCR does not always negatively correlate with expression in other teleosts, indicating that additional regulatory factors are present. This may account for the differences in LWS-cone λ_max _among guppy populations and individuals.

## Authors' contributions

CRJL and JST conceived and designed the experiments. Experiments, data compiling, and analysis were carried out by CRJL. The manuscript was written and illustrated by CRJL, and incorporates revisions by JST. JST supervised the study. All authors read and approved the final manuscript.

## Supplementary Material

Additional file 1 **Spectral characteristics of guppy light-dark cycle aquaria**. Illumination was provided by broad spectrum fluorescent bulbs (GE F32T8SP/65K), with relative spectral irradiance measured by a USB2000 spectrophotometer (Ocean Optics, Inc.) at the air to water boundary during the light phase. Click here for file

Additional file 2 **Cross-amplification controls for LWS primer specificity**. (**A**) Locus-specific primer (LSP) sets, detailed in the methods, for each *LWS *(*A180*, *P180*, *S180*, and *S180r*) were individually used in an attempt to amplify off of *LWS*-containing plasmids (1.0E07 copies/reaction) under the RT-qPCR conditions detailed in the methods. Although cloned opsin gene (pGEM::*LWS*) sequences are not full length (i.e., incomplete open reading frame, ORF) their respective primer site locations are shared (although different in sequence) among all four genes. Plasmid inserts were generated using primers shown in part C. Amplification and dissociation curves are shown with corresponding agarose gel-electrophoresis images of resultant amplicons. 1kb+ DNA ladder was used (Invitrogen^®^). All reactions were run in parallel. Note the gel image corresponding to the *S180r *primer panel is not the same dimensions as the others, as lane gaps were not used during gel loading. (**B**) NAC and NTC controls are shown. (**C**) Table of Oligonucleotide primers and sequences used to generate LWS plasmids for cross-amplification experiments. Click here for file

Additional file 3 **Amplification efficiency differences among visual opsins**. qPCR plasmid standard-curve values used to assess differences in amplification efficiencies among six of the 10 visual-opsin gene constructs. Click here for file

Additional file 4 **Reference gene copy number for primary (A) and secondary (B) surveys**. Transcript copy number of *COI*, *β-actin*, and *Myosin-HC *reference genes determined by RT-qPCR analysis of guppy cDNA samples. (L) and (R) denote left and right eyes, respectively. Error bars (±S.E.M. of triplicate reactions) are shown for all samples, though most are too small to see. Click here for file

Additional file 5 **Normalization to multiple reference genes**. Justification of reference genes used for normalization. Click here for file
