# Supplementary material for: RT-qPCR reveals opsin gene upregulation associated with age and sex in guppies (Poecilia reticulata) - a species with color-based sexual selection and 11 visual-opsin genes
Source: BMC Evol Biol. 2011 Mar 29;11:81. doi: 10.1186/1471-2148-11-81 (PMC3078887; doi:10.1186/1471-2148-11-81)
Supplement: Additional file 2 — Cross-amplification controls for LWS primer specificity. (A) Locus-specific primer (LSP) sets, detailed in the methods, for each LWS (A180, P180, S180, and S180r) were individually used in an attempt to amplify off of LWS-containing plasmids (1.0E07 copies/reaction) under the RT-qPCR conditions detailed in the methods. Although cloned opsin gene (pGEM::LWS) sequences are not full length (i.e., incomplete open reading frame, ORF) their respective primer site locations are shared (although different in sequence) among all four genes. Plasmid inserts were generated using primers shown in part C. Amplification and dissociation curves are shown with corresponding agarose gel-electrophoresis images of resultant amplicons. 1kb+ DNA ladder was used (Invitrogen®). All reactions were run in parallel. Note the gel image corresponding to the S180r primer panel is not the same dimensions as the others, as lane gaps were not used during gel loading. (B) NAC and NTC controls are shown. (C) Table of Oligonucleotide primers and sequences used to generate LWS plasmids for cross-amplification experiments. [file 1471-2148-11-81-S2.DOC]

**A180 primers**

**P180 primers**

**pGEM::A180**

**pGEM::P180**

**pGEM::S180**

**Plasmid copy number per reaction = 1.0E07 (10 million)**

**LWS primer specificity testing**

**pGEM::S180r**


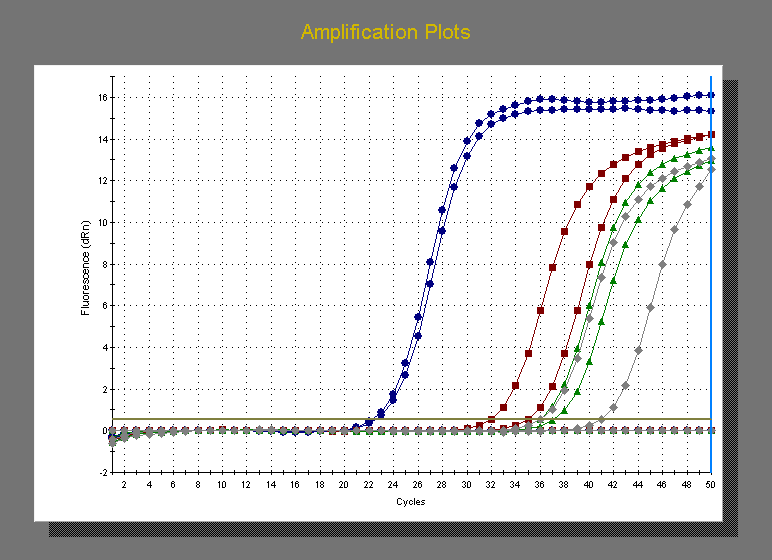

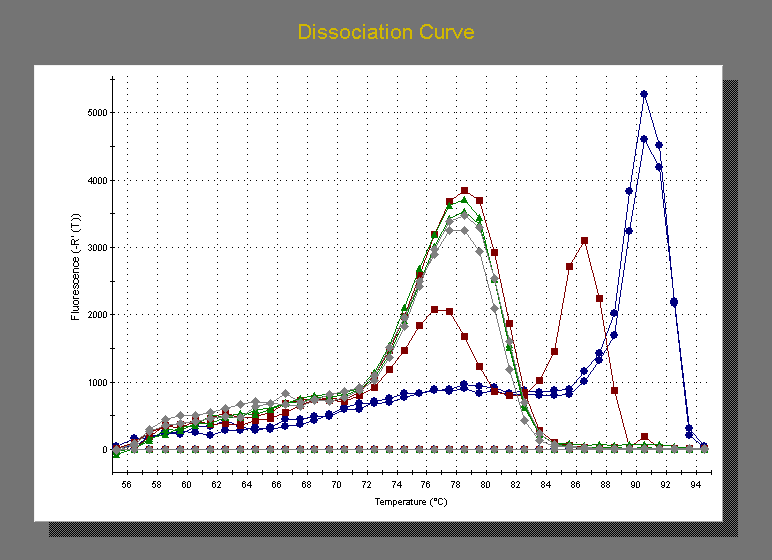

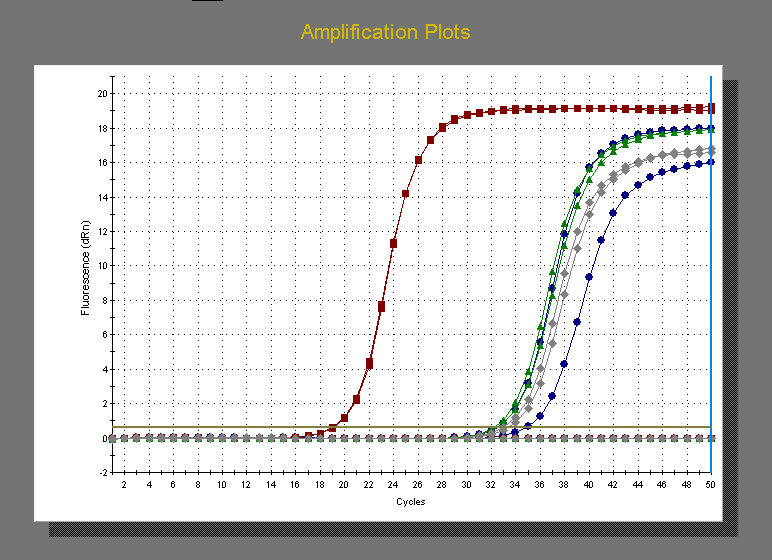

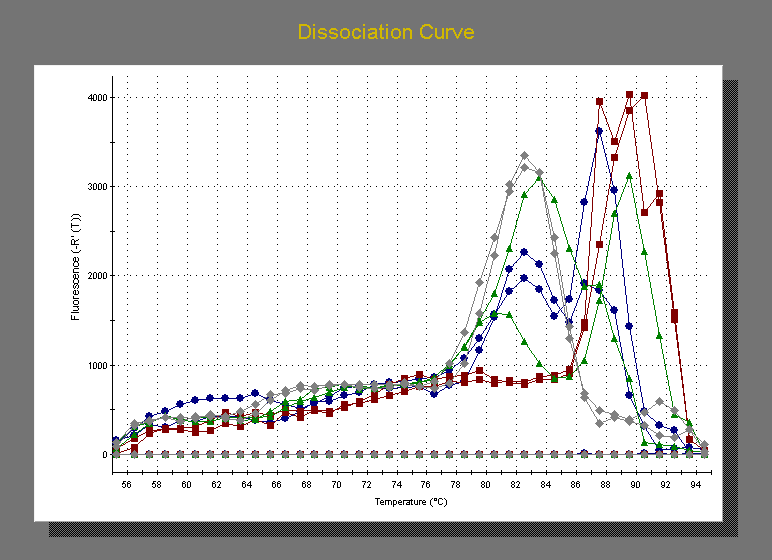


**Amplification curves**

**Dissociation curves**


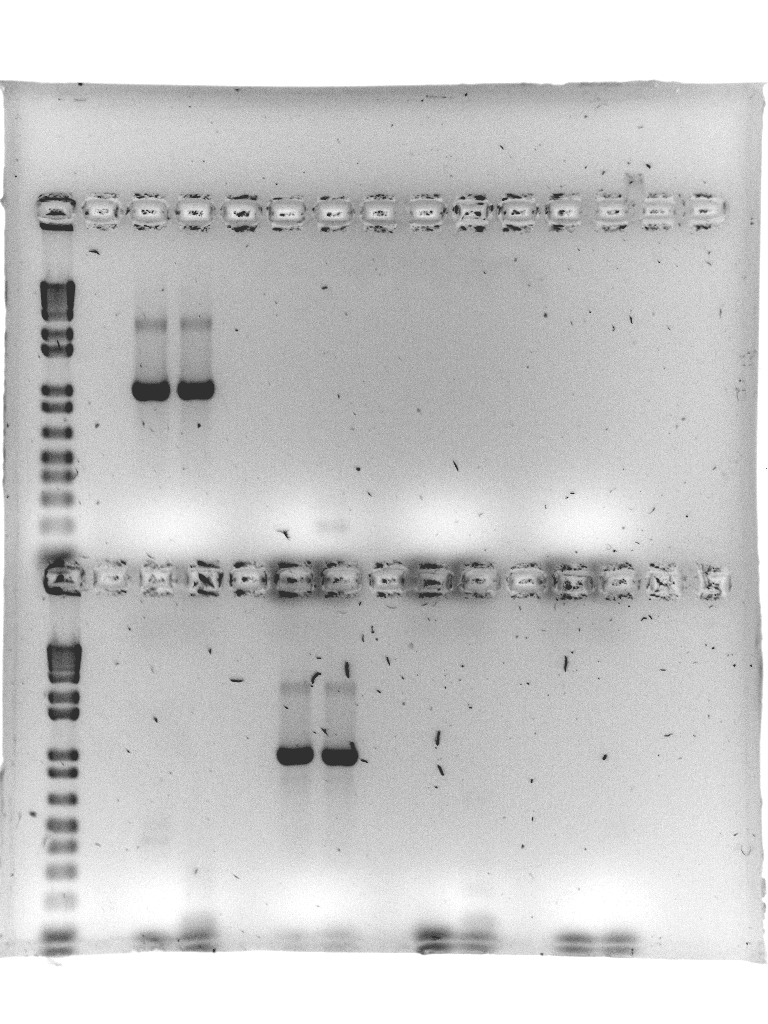


**p::A180**

**p::P180**

**p::S180**

**1000bp**

**p::S180r**

**p::A180**

**p::P180**

**p::S180**

**1000bp**

**p::S180r**

**S180 primers**

**S180r primers**


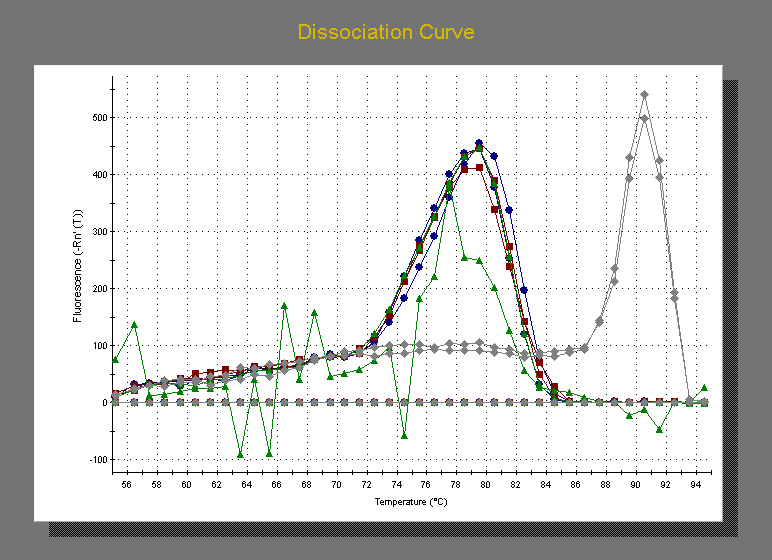

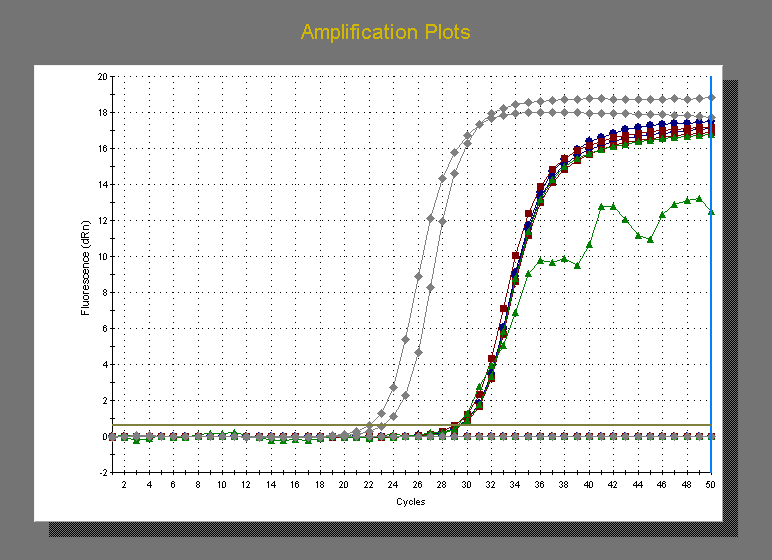

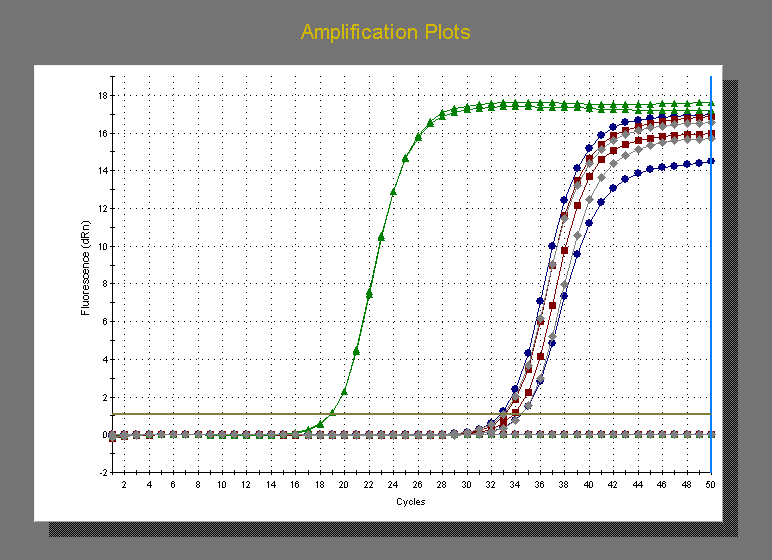

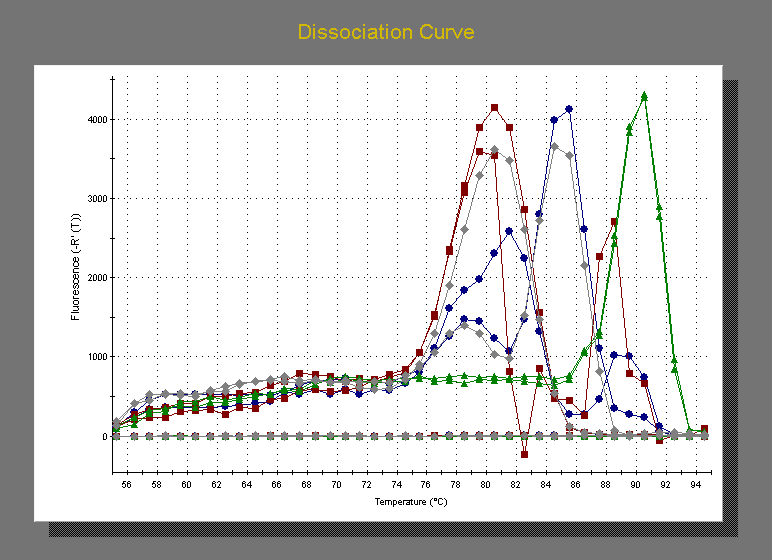

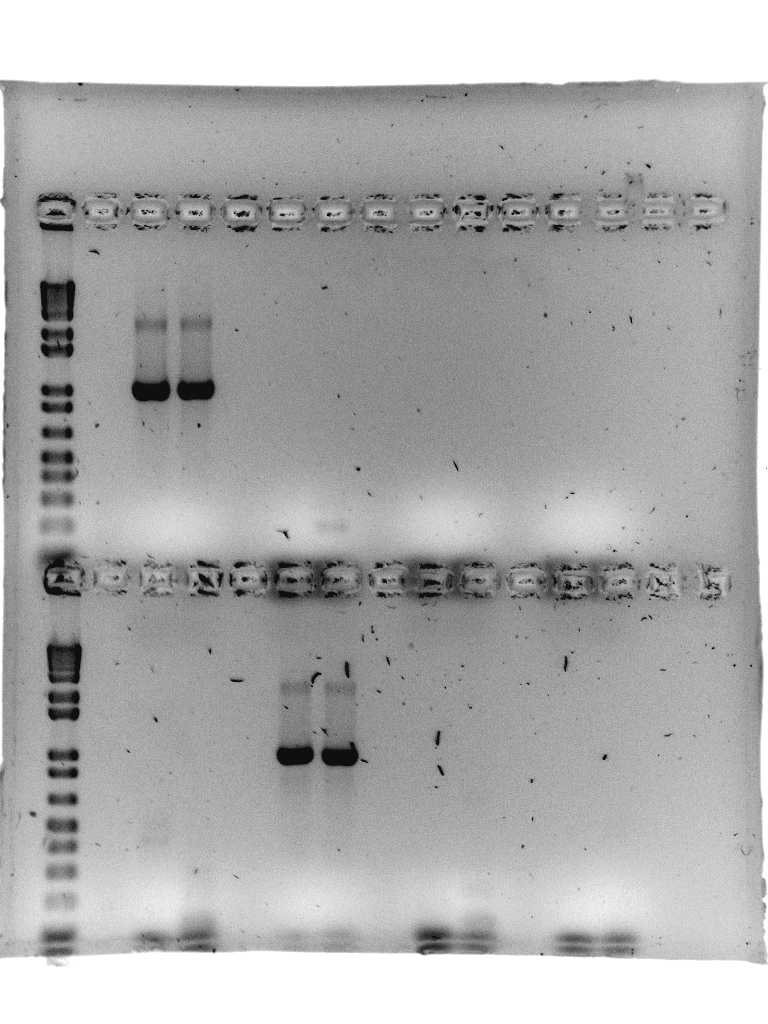

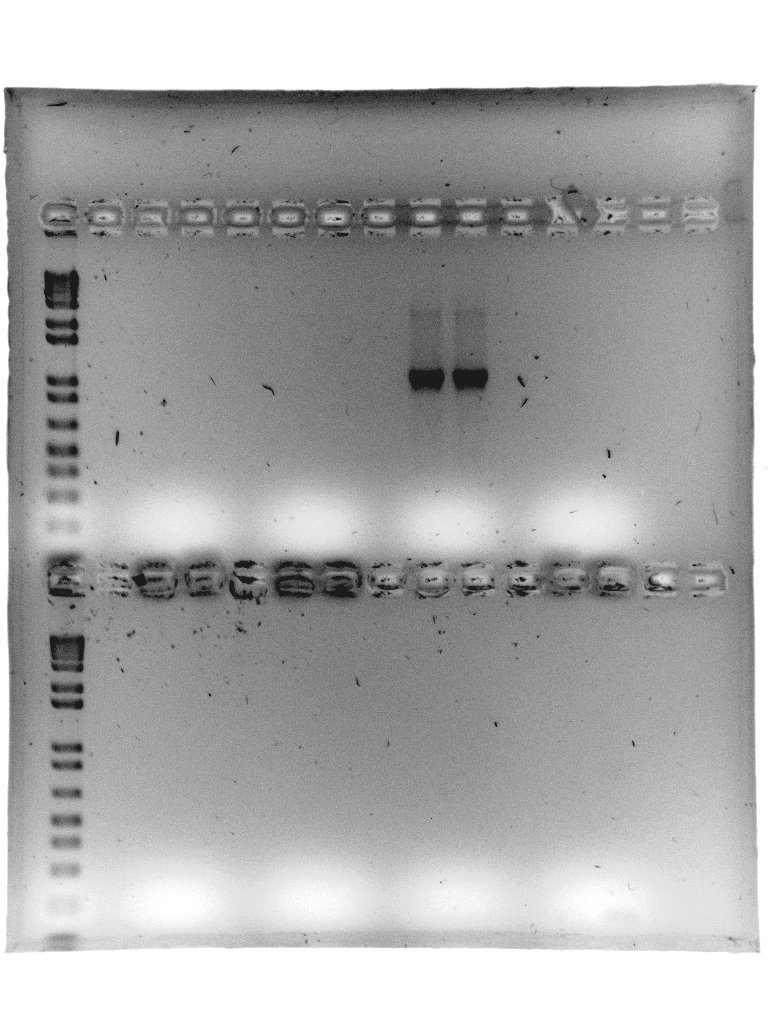


**p::A180**

**p::P180**

**p::S180**

**1000bp**

**p::S180r**


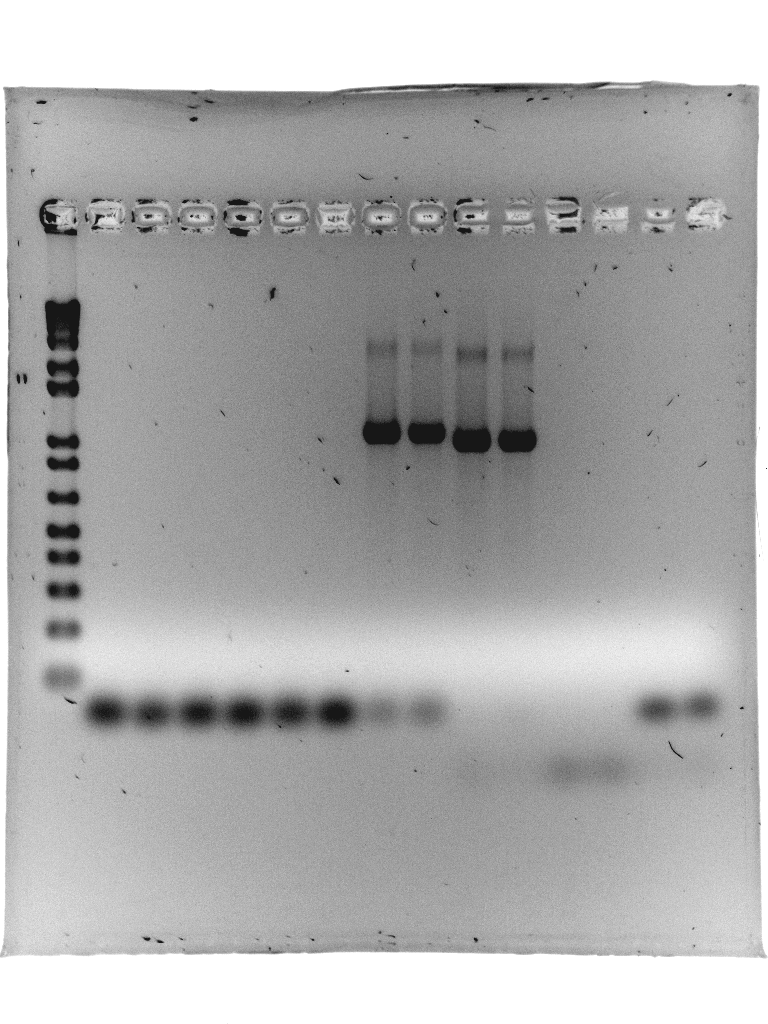


**p::A180**

**p::P180**

**1000bp**

**p::S180r**

**p::S180**

**Color code**

**A**

**NAC**

**LWS primer specificity testing (controls)**

**NTC**


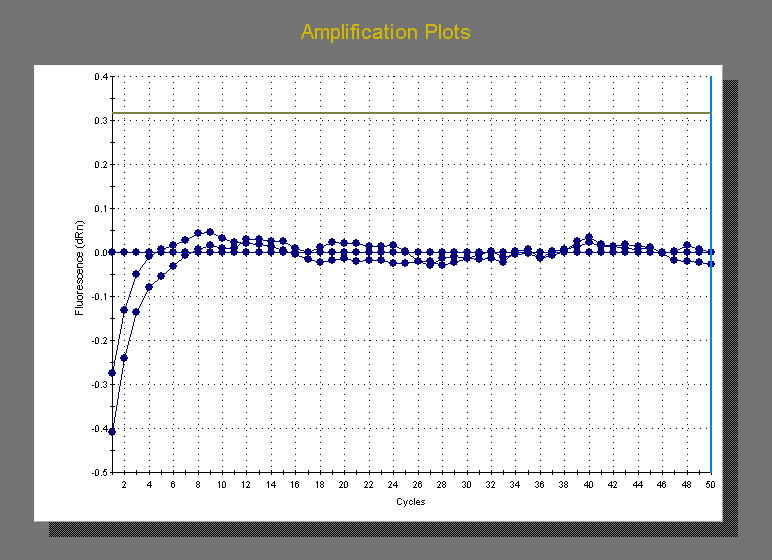

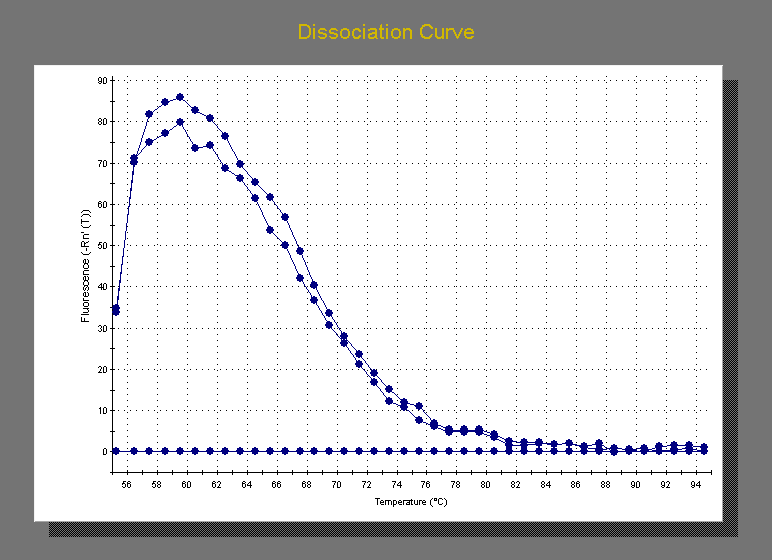

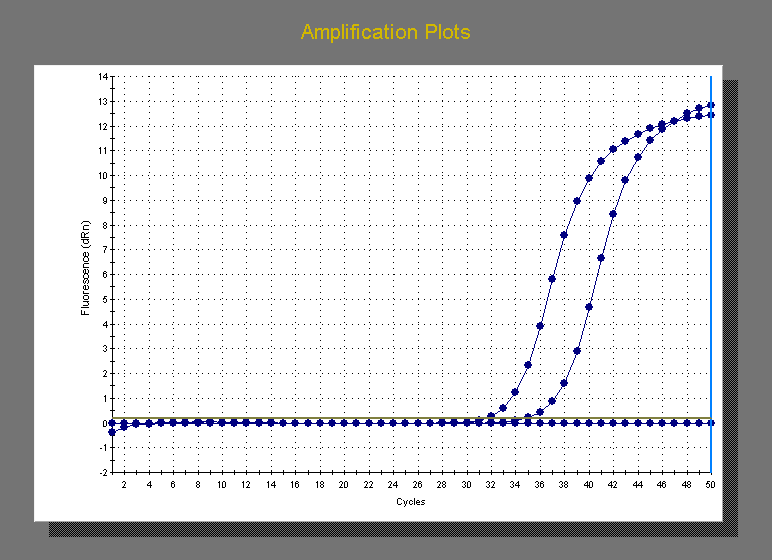

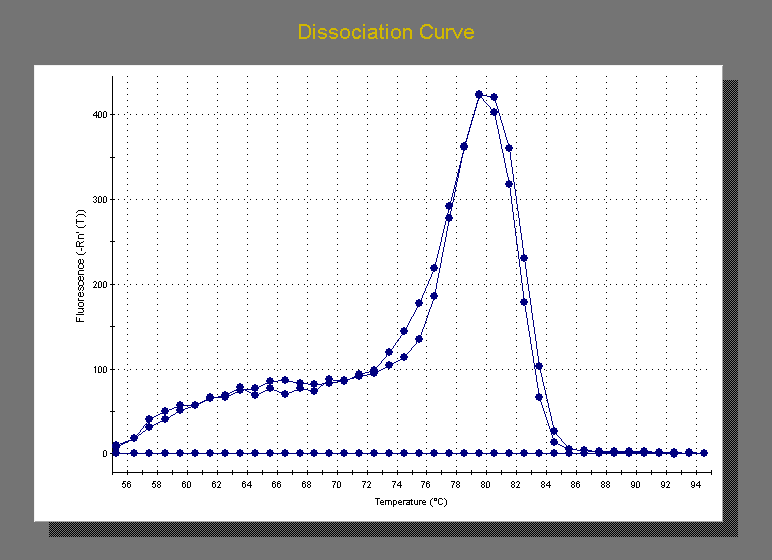

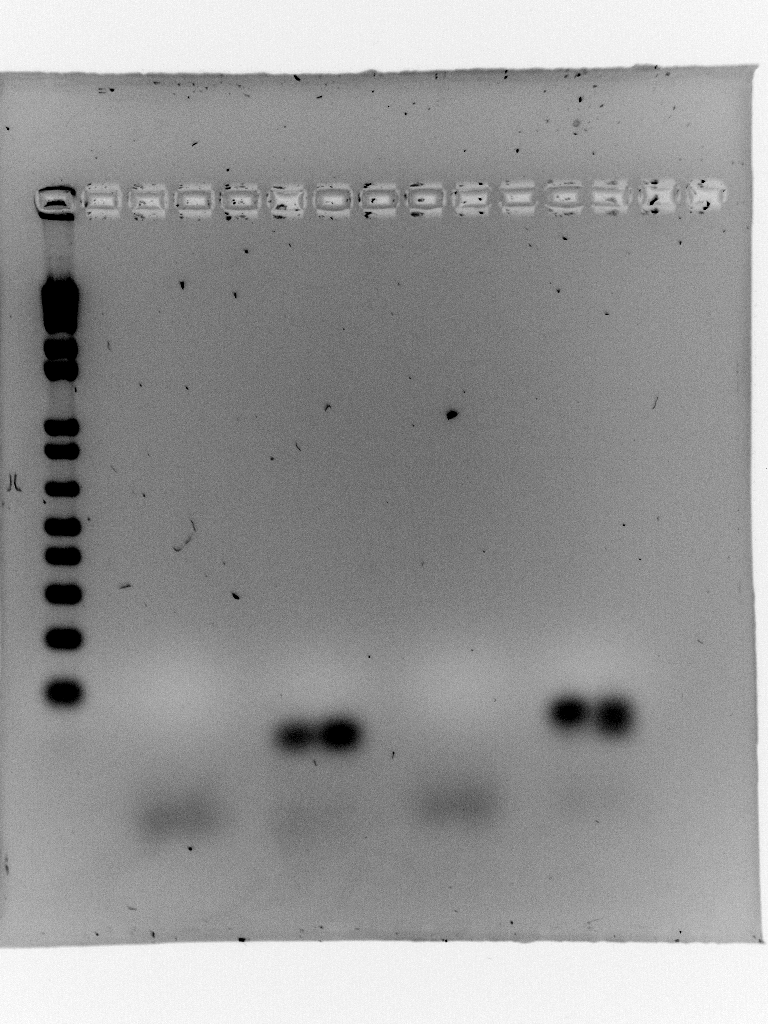


**1000bp**

**NAC**

**NTC**

**Amplification curves**

**Dissociation curves**

**B**

**C**)

| ***Visual*** ***opsin***  ***gene***  ***insert*** | ***Primer Name*** | ***Primer Sequence (5’→3’)*** | ***Amplicon/insert size (bp) and GenBank accession***  ***number*** | ***Primers designed against GenBank accession***  ***number or reference*** |
| --- | --- | --- | --- | --- |
| *LWS A180^a^* | ForBeg | ATGGCAGAGGAATGGGGAAAAC | (1166)  HQ260675 | [8] |
|  | A1803’UTR-R | ATAGGGCCCGCAATGCAAC |  | DQ168659 |
| *LWS P180^a^* | Fw100 | GATCCCTTTGAAGGACCAAACT | (1057)  HQ260676 | [8] |
|  | LWS2 IntRev | CAGTCCCGGCAGTAATAACAAAC |  |  |
| *LWS S180^a^* | ForBeg | ATGGCAGAGGAATGGGGAAAAC | (1168)  HQ260677 | [8] |
|  | LWS1 IntRev | CATTTGCCATAAAGTTTCCGTTTATC |  |  |
| *LWS S180r^a^* | S180rEx1-Ex2span-S | GCAATCATACAAGAGATCC | (1181)  HQ260678 | Watson et al., unpublished data |
|  | S180r-3’UTR-R | GACAACGGGGAGTATTGGTG |  |  |
