# Supplementary material for: RT-qPCR reveals opsin gene upregulation associated with age and sex in guppies (Poecilia reticulata) - a species with color-based sexual selection and 11 visual-opsin genes
Source: BMC Evol Biol. 2011 Mar 29;11:81. doi: 10.1186/1471-2148-11-81 (PMC3078887; doi:10.1186/1471-2148-11-81)
Supplement: Additional file 3 — Amplification efficiency differences among visual opsins. qPCR plasmid standard-curve values used to assess differences in amplification efficiencies among six of the 10 visual-opsin gene constructs. [file 1471-2148-11-81-S3.DOC]

| *Standard Curve* | *Linear Equation* | *Amplification Efficiency (%)* | *RSq* |
| --- | --- | --- | --- |
| *A180** | Y= -3.396*Log(X) + 41.42 | 97.0 | 0.985 |
| *P180* | Y= -3.691*Log(X) + 40.73 | 86.6 | 0.995 |
| *S180r** | Y= -2.944*Log(X) + 38.19 | 118.6 | 0.993 |
| *RH2-1* | Y= -3.807*Log(X) + 45.03 | 83.1 | 0.998 |
| *RH2-2* | Y= -3.685*Log(X) + 44.03 | 86.8 | 0.999 |
| *SWS2B* | Y= -3.862*Log(X) + 43.86 | 81.5 | 0.998 |

*** Primer sets used on these clones are not the same as those used for RT-qPCR in the present study, as they proved to be cross reactive (data not shown). Thus, new primers were designed and specificity tested (see Additional file 2).
