# Supplementary material for: RT-qPCR reveals opsin gene upregulation associated with age and sex in guppies (Poecilia reticulata) - a species with color-based sexual selection and 11 visual-opsin genes
Source: BMC Evol Biol. 2011 Mar 29;11:81. doi: 10.1186/1471-2148-11-81 (PMC3078887; doi:10.1186/1471-2148-11-81)
Supplement: Additional file 4 — Reference gene copy number for primary (A) and secondary (B) surveys. Transcript copy number of COI, β-actin, and Myosin-HC reference genes determined by RT-qPCR analysis of guppy cDNA samples. (L) and (R) denote left and right eyes, respectively. Error bars (±S.E.M. of triplicate reactions) are shown for all samples, though most are too small to see. [file 1471-2148-11-81-S4.DOC]

**A**)

**B**)
