# Supplementary material for: RT-qPCR reveals opsin gene upregulation associated with age and sex in guppies (Poecilia reticulata) - a species with color-based sexual selection and 11 visual-opsin genes
Source: BMC Evol Biol. 2011 Mar 29;11:81. doi: 10.1186/1471-2148-11-81 (PMC3078887; doi:10.1186/1471-2148-11-81)
Supplement: Additional file 5 — Normalization to multiple reference genes. Justification of reference genes used for normalization. [file 1471-2148-11-81-S5.DOC]

The RT-qPCR normalization strategy first implemented in this study was to equalize the total RNA input among reverse transcription (RT) reactions (described in methods). This normalization technique is limited in the sense that it cannot account for differences in RNA degradation, secondary structures, or other factors known to affect RT efficiency among reactions, which can cause expression-independent changes in cDNA concentration (thoroughly reviewed in references [1-3]). To compensate, normalization to multiple reference genes was carried out after transcript copy number for each opsin paralog was established. To do so, transcript copy number for *COI*, *β-actin*, and *Myosin-HC* were determined (as per the RT-qPCR conditions detailed in the methods). This resulted in similar expression profiles (see Additional file 4) for the 10 primary-survey and eight secondary-survey cDNA samples (Table 1), indicating that the differences in copy number among cDNA samples for a given gene, are due to one or more of the expression-independent changes in cDNA concentration described above, and not to endogenous changes in expression. These data were averaged (geometric mean) and used to normalize opsin gene transcript copy number, yielding fold-change in expression values relative to the reference genes.

Software that can assist in finding the most suitable reference gene(s), such as GenEx^TM^ (MultiD Analyses®), was not used in this study. This software can determine the gene with the least variation in expression among samples. However, the caveat of this technique is that RNA input must be of equal quality and quantity among reactions for this type of comparison. In the present study, we are uncertain of this, as a NanoDrop® was used to quantify total RNA; thus, there is the possibility of expression-independent changes to RNA, as mentioned above.

References

1. Bustin SA, Benes V, Garson JA, Hellemans J, Huggett J, Kubista M, Mueller R, Nolan T, Pfaffl MW, Shipley GL, et al: **The miqe guidelines: Minimum information for publication of quantitative real-time pcr experiments.** *Clin Chem* 2009, **55:**611-622.

2. Bustin SA, Benes V, Nolan T, Pfaffl MW: **Quantitative real-time rt-pcr--a perspective.** *J Mol Endocrinol* 2005, **34:**597-601.

3. Nolan T, Hands RE, Bustin SA: **Quantification of mrna using real-time rt-pcr.** *Nat Protoc* 2006, **1:**1559-1582.
