# Supplementary material for: RT-qPCR reveals opsin gene upregulation associated with age and sex in guppies (Poecilia reticulata) - a species with color-based sexual selection and 11 visual-opsin genes
Source: BMC Evol Biol. 2011 Mar 29;11:81. doi: 10.1186/1471-2148-11-81 (PMC3078887; doi:10.1186/1471-2148-11-81)
Supplement: Supplementary file 5 [file 1471-2148-11-81-S1.DOC]

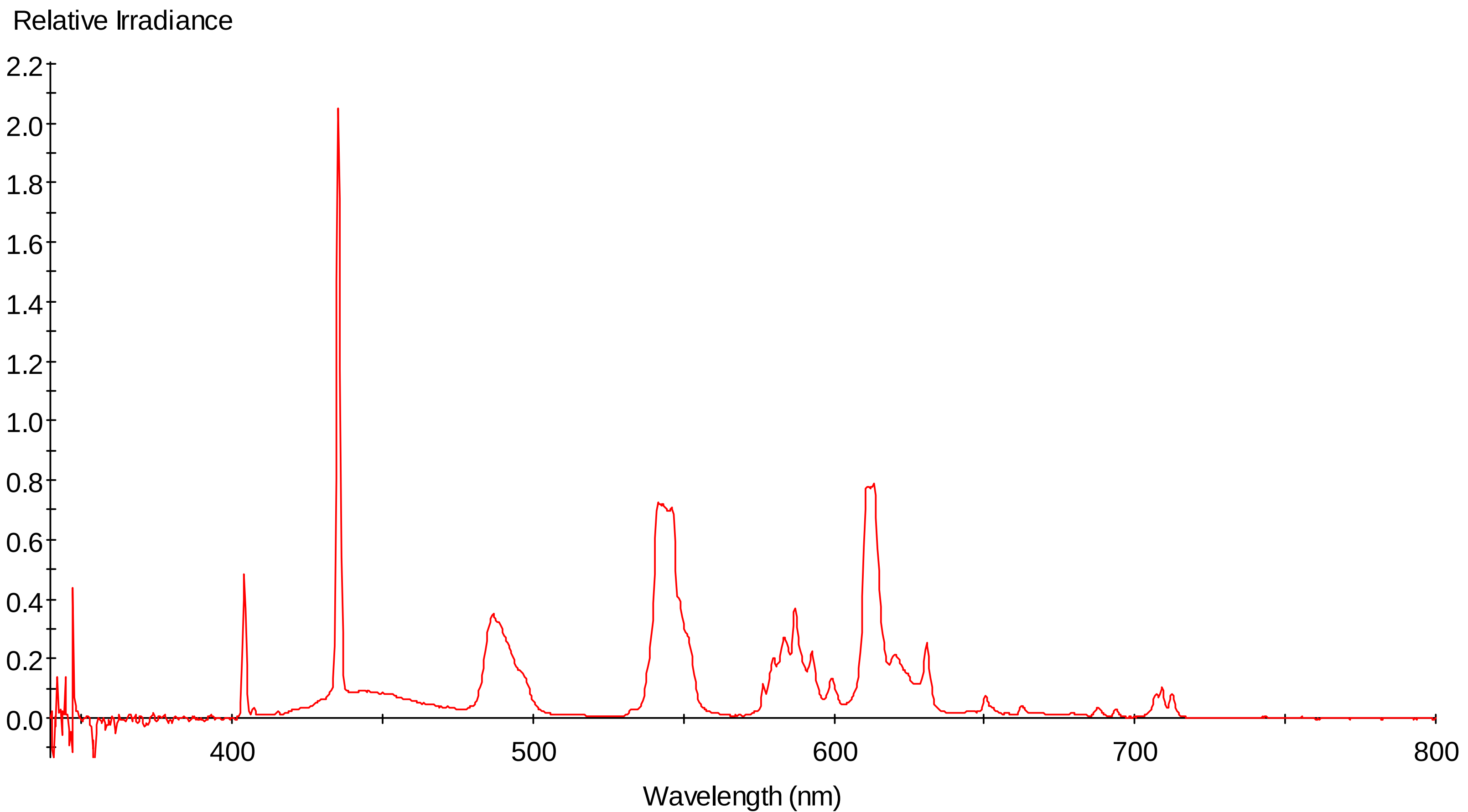
Relative Irradiance
2.2
2.0
1.8
1.6
1.4
1.2
1.0
0.8
0.6
0.4
0.2
0.0
400
500
600
700
800
Wavelength (nm)
